# Supplementary material for: Decreased but diverse activity of cortical and thalamic neurons in consciousness-impairing rodent absence seizures
Source: Nat Commun. 2023 Jan 10;14:117. doi: 10.1038/s41467-022-35535-4 (PMC9832004; doi:10.1038/s41467-022-35535-4)
Supplement: Supplementary file 1 — Supplementary Information [file 41467_2022_35535_MOESM1_ESM.pdf]

Supplementary Figure 1

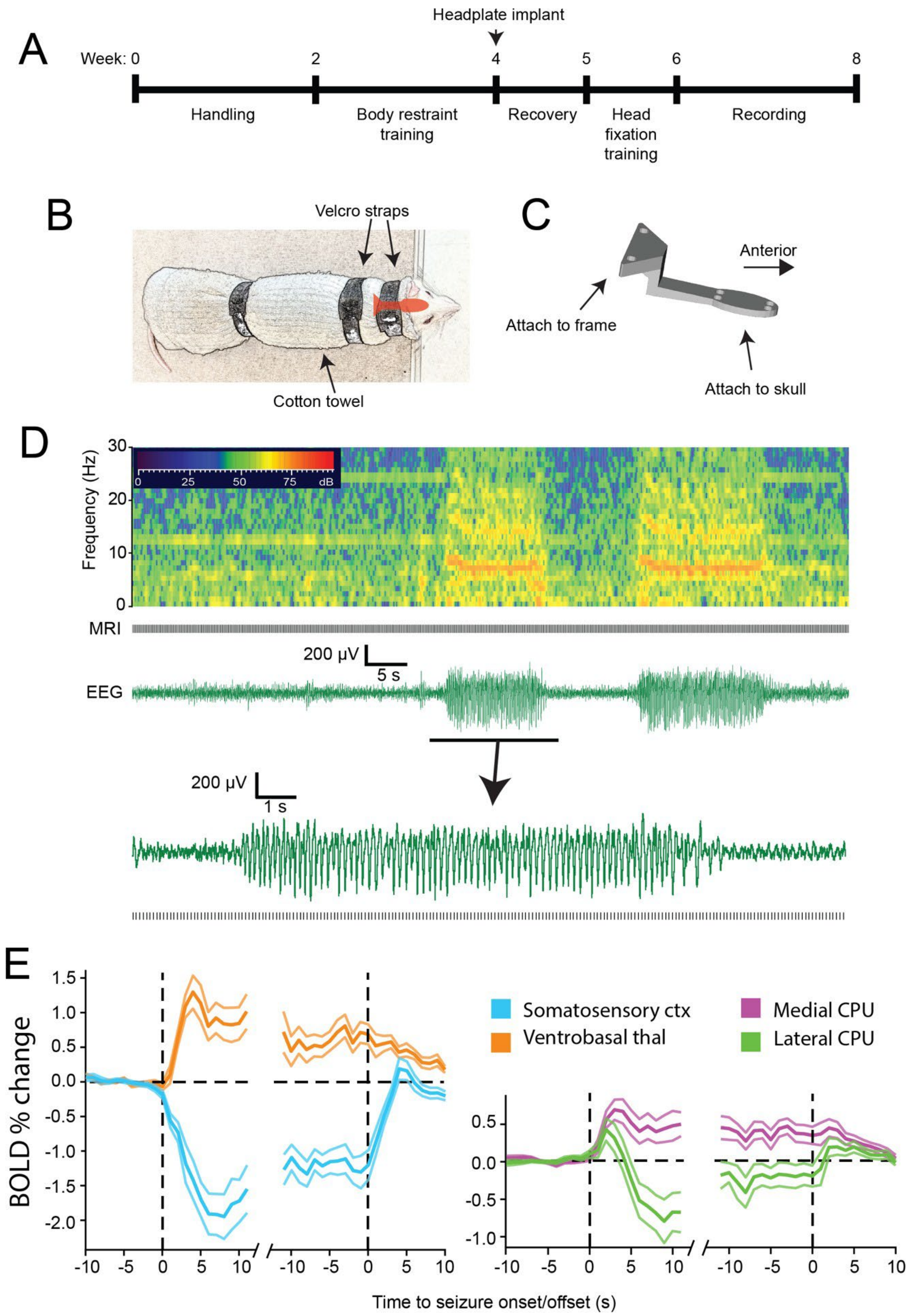

Supplementary Figure 1. Body & head restraint habituation and EEG/fMRI simultaneous acquisition. **A:** Experimental timeline for all fMRI data. **B:** Stylized image of GAERS rat in body restraint setup, with straps and towel carefully positioned to avoid uncomfortable torsion or painful constraint of limbs or other body parts. Orange shaded area shows general location of headplate after implantation. **C:** Schematic of 3D-printed MRI-compatible resin headplate used to connect to rat skull (permanently via dental cement) and to fMRI frame (temporarily during data acquisition via polycarbonate screws). **D:** Spectrogram (upper) and corresponding voltage time course (lower) of example EEG acquired via carbon filaments with simultaneous fMRI acquisition (indicated by markers of TTL pulses at each slice time (12 slices per second). Expanded EEG shows clearly identifiable spike-wave discharge during simultaneous fMRI acquisition, after application of smoothing and DC remove processes to remove scanning noise (see Methods). Corresponding spectrogram shows characteristic power changes with each SWD. Spectrogram color scale is in dB, calculated using the “sonogram” channel draw mode in Spike2 with Hanning window, range 0-96 dB, and a window size of 1024 samples (with 1023 sample overlap) per fast Fourier transform. **E:** Mean percent change timecourses of BOLD signals (± SEM) in the same regions as Fig. 1 aligned to SWD onset and offset in 1 s time bins, but with pre-seizure baseline set as 8 to 3 seconds before initiation, showing similar changes to those observed at the default baseline of 5 to 0 seconds (compare to Fig. 1 C).

Supplementary Figure 2

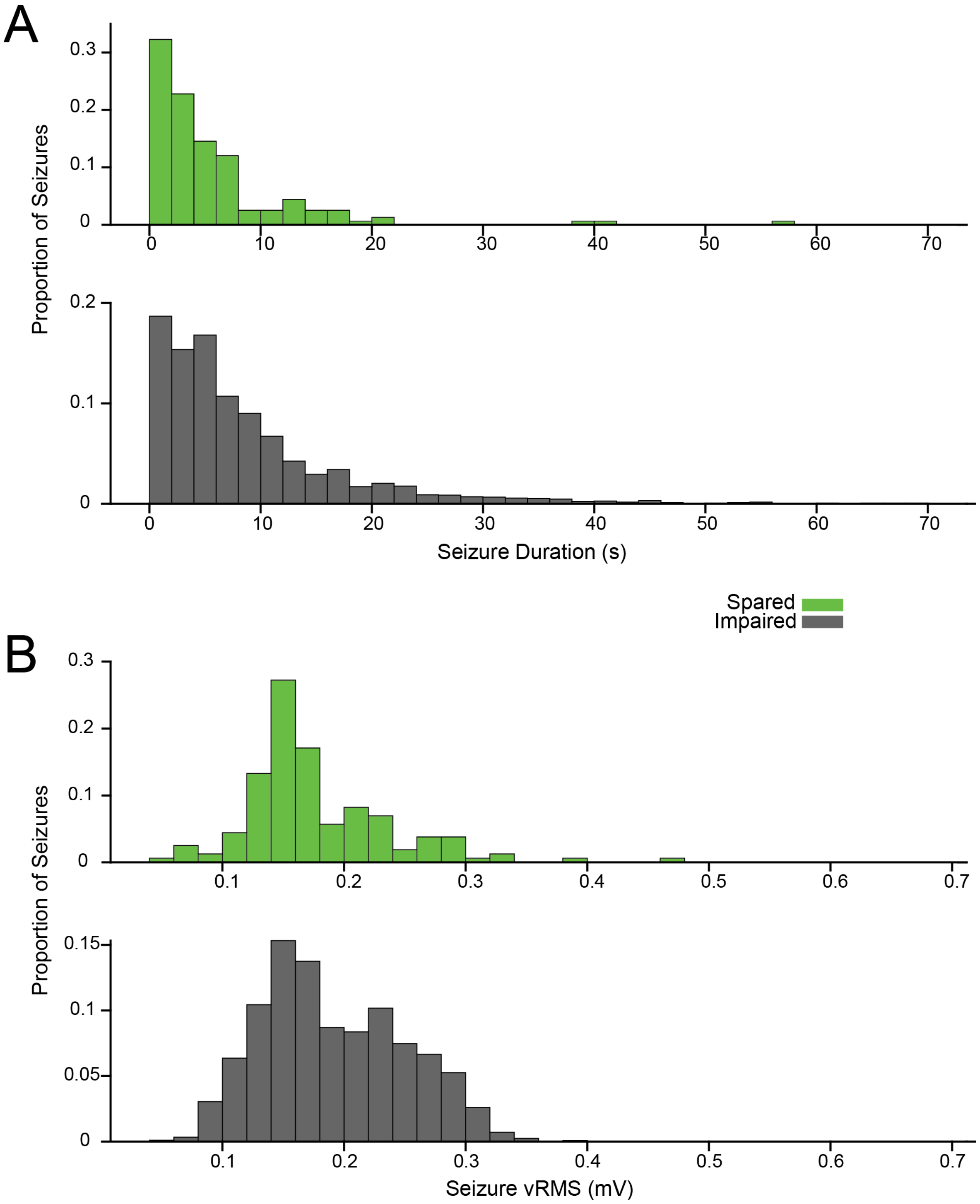

Supplementary Figure 2. Durations and vRMS amplitude of behaviorally spared (at least one lick) and impaired (no licks) SWDs. **A:** Normalized probability distribution (2 s bin width) of durations of spared (upper, green, n = 158) and impaired (lower, grey, n = 2988) SWDs. **B:** Normalized probability distribution (0.02 mV bin width) of vRMS amplitudes of the same two groups of SWDs.

Supplementary Figure 3

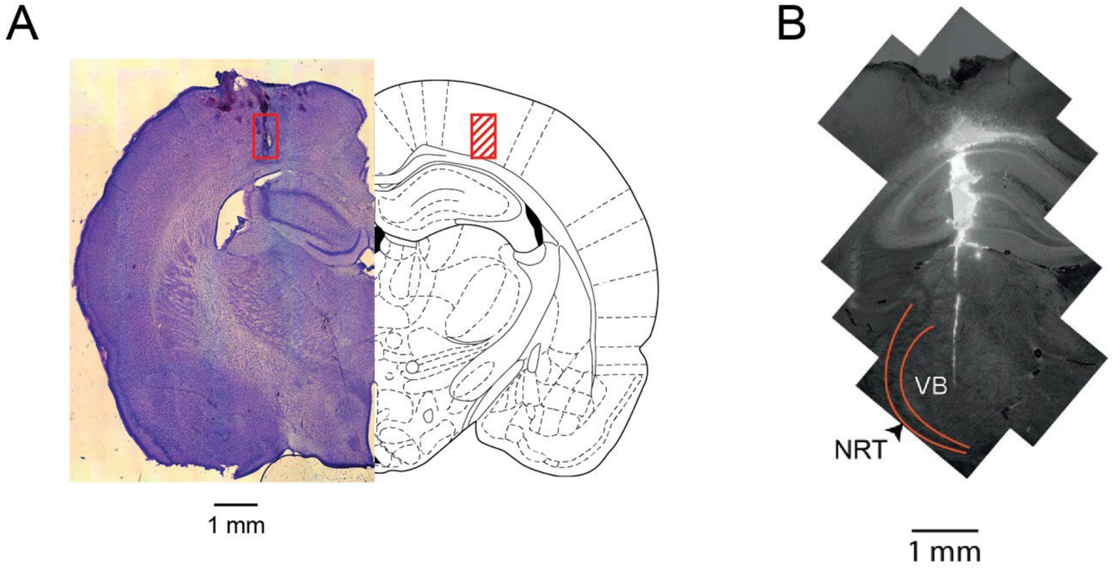

Supplementary Figure 3. Histology and locations of electrodes in cortex and thalamus **A:** sample histological section (on left) aligned with rat brain coronal section (on right) at bregma -3.6 mm (reproduced with permission from Paxinos & Watson Rat Brain Atlas, 2013[1], Copyright Elsevier) indicating target location and recording range (brain surface -0.9 to brain surface -2 mm) of primary somatosensory trunk cortical (S1Tr) electrode arrays. This was repeated for the 5 independent animals. **B:** Sample histological section showing the final position of a silicon probe in the ventrobasal thalamus (reproduced with permission from McCafferty et al., 2018[2], repeated for 8 independent animals).

Supplementary Figure 4

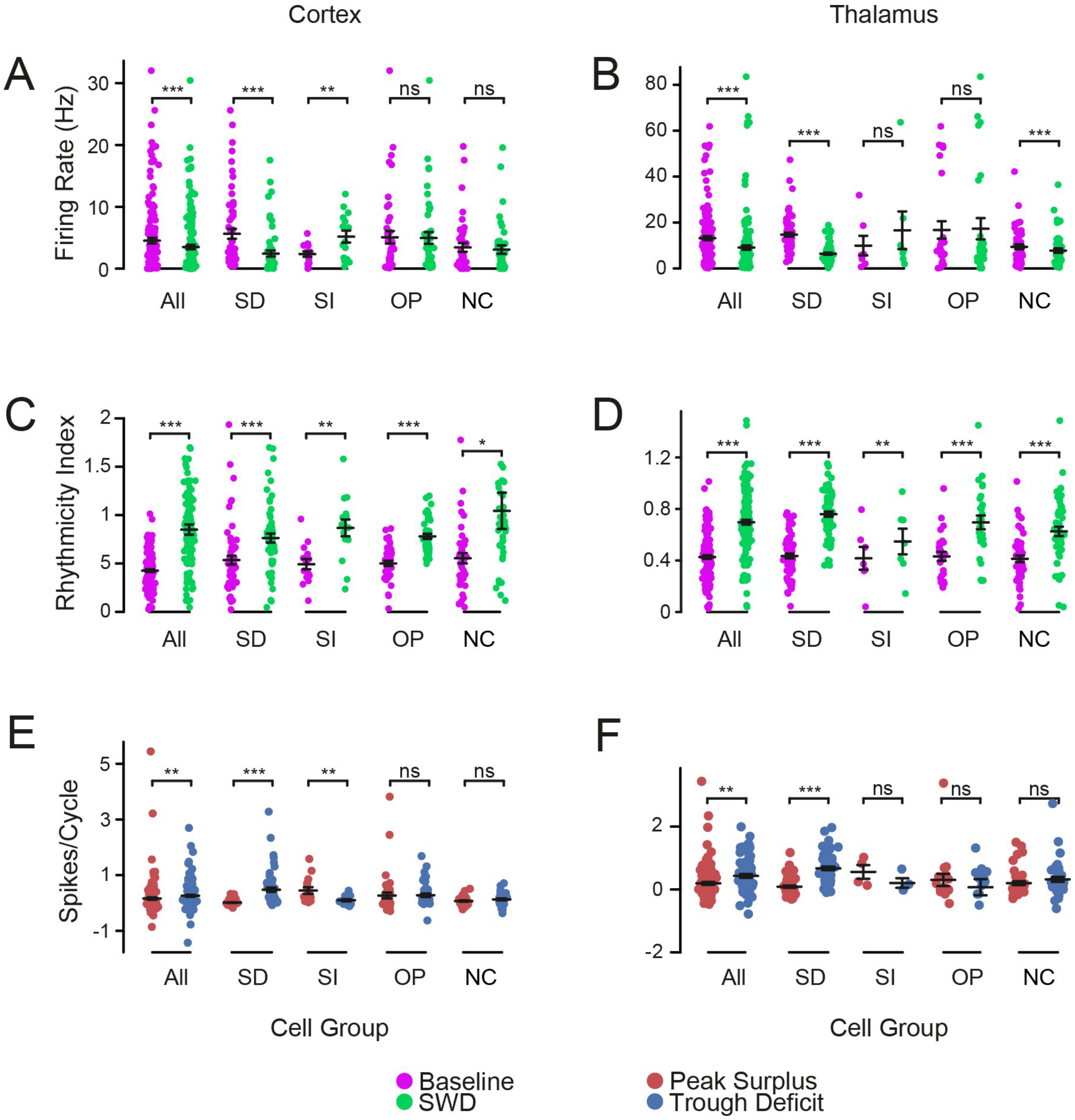

Supplementary Figure 4. Firing characteristics of cortical and thalamic neurons and subgroups at baseline and during SWDs. "All" groups are the same as in Fig. 3, reproduced for ease of comparison. **A:** Mean firing rate of each subgroup of cortical neurons at pre-seizure baseline (from 10 s to 5 s before seizure initiation) and during seizure, showing that the overall significant decrease during seizure in All neurons ($p = 0.0001$) is driven primarily by the Sustained Decrease (SD) group ($p = 3.5 \times 10^{-7}$), with an opposing increase in the Sustained Increase (SI) group ($p = 0.005$), no change in the Onset Peak (OP) group ($p = 1$) or in the No Change (NC) group ($p = 0.15$). **B:** As for **A** but for thalamic subgroups, showing the same decrease in firing overall ($p = 5.4 \times 10^{-12}$) and in the SD group ($p = 3.2 \times 10^{-21}$), with the SI group failing to reach significance ($p =0.66$; small sample size), no significant change in the OP group ($p = 1$), and a smaller decrease in NC ($p = 0.0002$). **C:** Mean rhythmicity (2 s bins) of all cortical neurons and each subgroup of cortical neurons in baseline and during SWDs. Baseline is defined here as all periods more than 5 s from the start or end of seizure. All groups show a significant increase in rhythmicity (All $p = 2.3 \times 10^{-9}$, SD $p = 6.4\times 10^{-5}$, SI $p = 0.003$, OP $p = 7.8 \times 10^{-11}$, NC $p = 0.04$). **D:** As for **C** but for thalamic neurons, showing the same unanimous increase in rhythmicity (All $p = 8.9 \times 10^{-17}$, SD $p = 1.02 \times 10^{-25}$, SI $p = 0.0024$, OP $p = 0.0002$, NC $p = 1.8 \times 10^{-10}$). **E:** Mean firing for each cortical neuron during defined peak and trough periods during spike-wave cycles, relative to firing during matched periods during baseline (all periods more than 5 s from start or end of seizure, see Methods for details). A larger trough deficit than peak surplus in the Sustained Decrease group ($p = 1.8 \times 10^{-6}$) explains the overall dominance of trough deficits ($p = 0.006$), while the peak surplus is larger in the Sustained Increase group ($p = 0.02$). **F:** as for **E** but for thalamic neurons, showing the same overall ($p = 0.007$) and Sustained Decrease ($p = 6.5 \times 10^{-10}$) differences. Central lines indicate mean and whiskers indicate standard error of the mean. Total number

of neurons (n) in analyses are for cortex All 165, SD 59, SI 15, OP 44, NC 41; and for thalamus All 163, SD 76, SI 7, OP 27, NC 52. *** $p < 0.001$; ** $p < 0.01$; * $p < 0.05$; ns, not significant. All statistical tests were two-sided paired t-tests, with Bonferroni correction for multiple comparisons.

Supplementary Figure 5

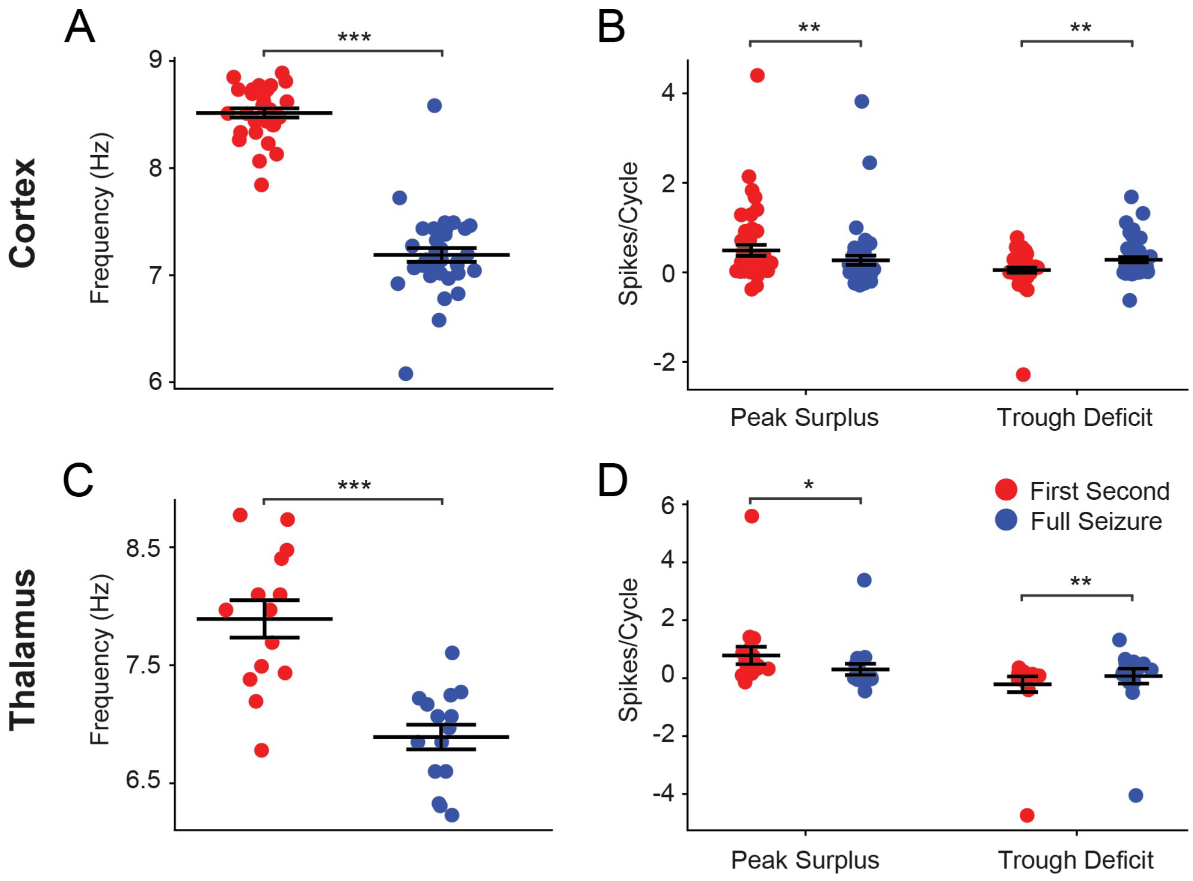

Supplementary Figure 5. Firing characteristics of cortical (n = 32 cells) and thalamic (n = 18 cells) OP neurons during the first second and full seizure. **A:** Oscillation frequency (inverse of peak-to-peak interval) of cortical neurons is higher during the first second of seizure ($p = 4.5 \times 10^{-16}$). **C:** Same as for **A** but for thalamic neurons ($p = 4.9 \times 10^{-7}$). **B:** Mean firing surplus for cortical neurons during defined peak periods of spike-wave cycles (relative to firing during matched periods of baseline) is greater during the first second of seizure (p = 0.016) while mean firing deficit during trough periods (relative to the same baselines) is smaller (p = 0.007). **D:** Same as for **B** but for thalamic neurons (peak surplus p = 0.0005; trough deficit p = 0.005). *** $p < 0.001$; ** $p < 0.01$. All error bars represent SEM. All statistical tests are two-sided paired t-tests.

References

1. Paxinos G, Watson C. *The Rat Brain in Stereotaxic Coordinates*, 7th edn. Academic Press (2013).

2. McCafferty C*, et al.* Cortical drive and thalamic feed-forward inhibition control thalamic output synchrony during absence seizures. *Nat Neurosci* **21**, 744-756 (2018).
